# Cortical Binding Potential of Opioid Receptors in Patients With Fibromyalgia Syndrome and Reduced Systemic Interleukin-4 Levels – A Pilot Study

**DOI:** 10.3389/fnins.2020.00512

**Published:** 2020-05-19

**Authors:** Nurcan Üçeyler, Hans-Georg Buchholz, Susanne Kewenig, Stephan-Johann Ament, Frank Birklein, Mathias Schreckenberger, Claudia Sommer

**Affiliations:** ^1^Department of Neurology, University of Würzburg, Würzburg, Germany; ^2^Department of Nuclear Medicine, University of Mainz, Mainz, Germany; ^3^Department of Neurology, University of Mainz, Mainz, Germany

**Keywords:** fibromyalgia syndrome, PET, brain, opioid, IL-4

## Abstract

**Objective:** We investigated cerebral opioid receptor binding potential in patients with fibromyalgia syndrome (FMS) using positron-emission-tomography (PET) and correlated our results with patients’ systemic interleukin-4 (IL-4) gene expression.

**Methods:** In this pilot study, seven FMS patients (1 man, 6 women) agreed to participate in experimental PET scans. All patients underwent neurological examination, were investigated with questionnaires for pain, depression, and FMS symptoms. Additionally, blood for IL-4 gene expression analysis was withdrawn at two time points with a median latency of 1.3 years. Patients were investigated in a PET scanner using the opioid receptor ligand F-18-fluoro-ethyl-diprenorphine ([18F]FEDPN) and results were compared with laboratory normative values.

**Results:** Neurological examination was normal in all FMS patients. Reduced opioid receptor binding was found in mid cingulate cortex compared to healthy controls (*p* < 0.005). Interestingly, three patients with high systemic IL-4 gene expression had increased opioid receptor binding in the fronto-basal cortex compared to those with low IL-4 gene expression (*p* < 0.005).

**Conclusion:** Our data give further evidence for a reduction in cortical opioid receptor availability in FMS patients as another potential central nervous system contributor to pain in FMS.

## Introduction

There is an ongoing discussion on potential contributions of the peripheral and central nervous system (PNS, CNS) to the pathophysiology of pain in fibromyalgia syndrome (FMS). The CNS has been investigated with different methods pointing to an altered processing of nociceptive input in FMS patients compared to controls. Current evidence supports increased central sensitization, morphological alterations in cortical areas, reduced functional connectivity of descending pathways, and increased activity in the cerebral “pain matrix” (Cagnie et al., 2014; Dehghan et al., 2016). In a recent study, even a potential influence of central mechanisms on peripheral innervation was suggested in an experimental model (Harte et al., 2017).

Reduction in central opioid receptor binding has been described in FMS patients (Harris et al., 2007) and was confirmed in antinociceptive brain regions (Schrepf et al., 2016). In a previous study, we showed reduced systemic gene and protein expression of the anti-inflammatory and analgesic cytokine interleukin-4 (IL-4) in patients with FMS compared to healthy controls (Üçeyler et al., 2006). IL-4 links the neuro-immune and the opioid system by inducing μ-opioid receptor gene transcription (Kraus et al., 2001). We hypothesized that FMS patients with low systemic IL-4 gene expression might also have a reduced central opioid binding capacity, potentially leading to low opioid-sensitivity to endogenous and exogenous opioids as a contributor to FMS pain.

## Subjects and Methods

### Subjects

Our study was approved by the Ethics Committees of the Universities of Würzburg and Mainz, Germany. Written informed consent was obtained from all patients before inclusion. Patients were recruited at the Department of Neurology, University of Würzburg between 2007 and 2010. We enrolled seven opioid naïve FMS patients (1 man, 6 women) with a median age of 58 years (51–69 years) fulfilling the American College of Rheumatology 1990 diagnostic criteria (Wolfe et al., 1990) and who were willing to undergo experimental PET assessment in addition to the tests performed to investigate the PNS (Üçeyler et al., 2013) and the CNS (Üçeyler et al., 2015). Except for one patient, all patients were seen at three visits (Supplementary Table S1). Inclusion criteria were: men and women ≥ 18 years, other possible differential diagnoses excluded (e.g., rheumatologic, orthopedic), no clinically relevant psychiatric disorder (examined by systematic psychiatric interview). Exclusion criteria were: pain of other origin than FMS (e.g., post-surgery pain), current or prior cerebral diseases (e.g., stroke, cerebral hemorrhage or head trauma), prior or current opioid intake.

### Clinical Examination and Questionnaire Assessment

Patients underwent complete neurological examination at the Department of Neurology, University of Würzburg, Germany by the same investigator (N.Ü.) and the diagnosis of FMS was confirmed (Wolfe et al., 1990). For standardized pain assessment we used the German version of the Neuropathic Pain Symptom Inventory (NPSI) and the Graded Chronic Pain Scale (GCPS). To investigate depressive symptoms, the German version of the Beck Depression Inventory II (BDI) was applied. The calculated BDI scores reflect > 14 mild, >20 medium, >29 severe depressive symptoms. The German version of the Fibromyalgia Impact Questionnaire (FIQ) was used to determine FMS associated symptoms and their burden on overall well-being in the week before assessment. The FIQ was used instead of the revised FIQ, which was published after study initiation.

### Blood Withdrawal and IL-4 Gene Expression Analysis

Venous whole blood was withdrawn at 8–9 a.m. in monovettes containing ethylene-diamine-tetra-acetic acid after over-night fasting. A second blood sample was collected from six of the seven patients during a routine clinical follow-up visit (median latency 1.3 years after first visit) under the same conditions; of one patient blood could be obtained only during the second visit (see below). Aliquoted and flash-frozen (in liquid nitrogen) blood samples were stored at –80°C before further processing.

Gene expression analysis was performed as described earlier (Üçeyler et al., 2006). mRNA was isolated from frozen blood samples using a guanidine thiocyanate containing kit (Roti Quick Kit^®^, Germany). Afterward, 750 ng of mRNA were reverse transcribed into cDNA with TaqMan Reverse Transcription Reagents^®^ (Applied Biosystems, Germany) and 5 μL cDNA each were used for gene expression analysis of IL-4. TaqMan Universal Master Mix^®^ (Applied Biosystems, Germany) and specific oligonucleotide primers for IL-4 (ID: Hs00174122_m1) and the endogenous control 18sRNA (ID: Hs99999901_s1) (Applied Biosystems, Germany) were applied. Each qRT-PCR reaction plate contained a calibrator sample, which was the blood sample of the control person whose threshold cycle(Ct)-value was next to the calculated mean of all control samples specific for each IL-4. Results were compared with values obtained from seven healthy control blood samples previously collected in our laboratory (1 man, 6 women; median age 57 years, range 31–76). Samples were measured as triplicates and data were evaluated with the comparative ΔΔCt-method.

### PET Scans

#### Data Acquisition

Within 4 weeks after clinical examination and blood withdrawal, PET scans were performed under resting conditions at the Department of Nuclear Medicine, University of Mainz, Germany using a Siemens EXACT PET scanner (Siemens/CTI, Knoxville, YN, United States) and the subtype-non-selective opioidergic radioligand [F-18]fluoroethyl-diprenorphine ([18F]FEDPN) as previously described (Baumgartner et al., 2006). Dynamic emission imaging in 3D-mode started simultaneously with [18F]FEDPN injection (185 ± 6 MBq) and had a total duration of 94 min. The recordings were scatter and attenuation corrected and then reconstructed with filtered back projection (FBP) using Hanning-Filter with a filter width of 7.3 mM.

#### Data Assessment

Parametric images of the non-displaceable binding potential as a parameter of regional cerebral opioid receptor availability were computed using the non-invasive Logan Plot with the occipital cortex as reference region. Previously scanned eleven healthy subjects (5 men, 6 women; median age 42 years, range 30–50 years) served as a normal control group using the same acquisition, reconstruction, and quantitation protocols.

### Statistical Analysis

Statistical analysis of the non-normally distributed qRT-PCR data was performed using SPSS software version 24 (IBM, Ehningen, Germany), and the non-parametric Mann-Whitney-*U*-test was applied. Significance was assumed at *p* < 0.05. Statistical analysis of the PET data was performed using SPM8 implemented in Matlab 7.9 (MathWorks, Sherborn, MA, United States). The analysis included several steps. First, one-way analysis of covariance (ANCOVA), controlling for global [18F]FEDPN binding potential and followed by *post hoc* unpaired *t*-tests, was used to identify significant differences in binding potential between the normal healthy controls and the opioid naïve FMS patients. The resulting set of values for each contrast constituted a statistical parametric map of t statistics (SPMt). The SPMt values were transformed to the unit normal distribution SPMz. To correct for multiple comparisons, SPMt offers the method of small volume correction (SVC). We performed SVC for a number of 1,500 voxels leading to a corrected *p* < 0.05. The statistical parametric maps for intergroup comparisons were based on a threshold for uncorrected probability of *p* < 0.005.

## Results

### Clinical Findings

Neurological examination was normal in all patients at both visits. The Table 1 and Supplementary Table S2 summarize the group and individual characteristics of the study population at the second visit (i.e., prior to PET scan assessment). All patients had chronic and constant pain with a median current pain intensity of 5 (range 3–7) on a numeric rating scale (NRS) ranging from zero (no pain) to ten (worst pain).

**TABLE 1:** Clinical characteristics of study cohort.

M, F	1, 6
Age (years)	58 (51–69)
Disease duration (years)	36 (20–45)
NPSI sum score	24 (13–70)
Current pain intensity (GCPS)	4 (3–7)
Overall pain intensity (GCPS)	60 (57–73)
Impairmant due to pain (GCPS)	2 (0–4)
BDI score	11 (7–30)
FIQ sum score	48 (30–68)

*Median values are given with ranges in brackets. BDI, Beck Depression Inventory; F, female; FIQ, Fibromyalgia Impact Questionnaire; GCPS, Graded Chronic Pain Scale; M, male; NPSI, Neuropathic Pain Symptom Inventory.*

### Systemic IL-4 Gene Expression Remains Stable Over Time

**FIGURE 1:**
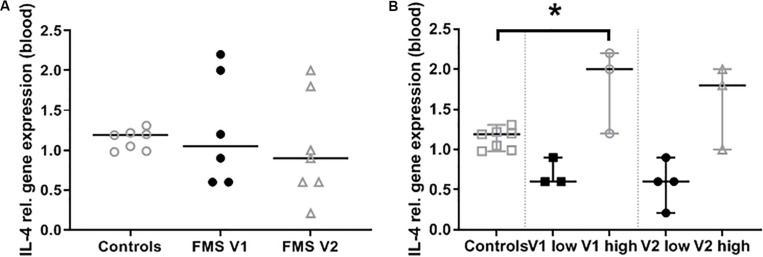
Interleukin-4 (IL-4) gene expression in blood samples. Graphs show the relative gene expression of IL-4 in whole blood samples of patients with fibromyalgia syndrome (FMS) compared to healthy controls. **(A)** Gene expression does not differ between visit 1 and 2. **(B)** At both visits the FMS group comprised patients with low and high IL-4 gene expression compared to healthy controls. The difference became significant at visit 1 comparing patients with high IL-4 expression with controls (*p < 0.05).

IL-4 gene expression was detectable in blood samples of all patients who had a blood withdrawal at both visits. Gene expression levels remained stable over time (Figure 1A). In an exploratory approach, we normalized the obtained qRT-PCR data of our healthy control group to “1.” Relative IL-4 gene expression in blood samples was < 1 (“low”) in four patients and > 1 (“high”) in three patients (Supplementary Table S3 and Figure 1B). PET scan data were stratified to the entire FMS patient group compared to laboratory normative values and for low and high IL-4 gene expression.

### Cerebral Opioid Binding Is Lower in FMS Patients Than in Healthy Controls

**FIGURE 2:**
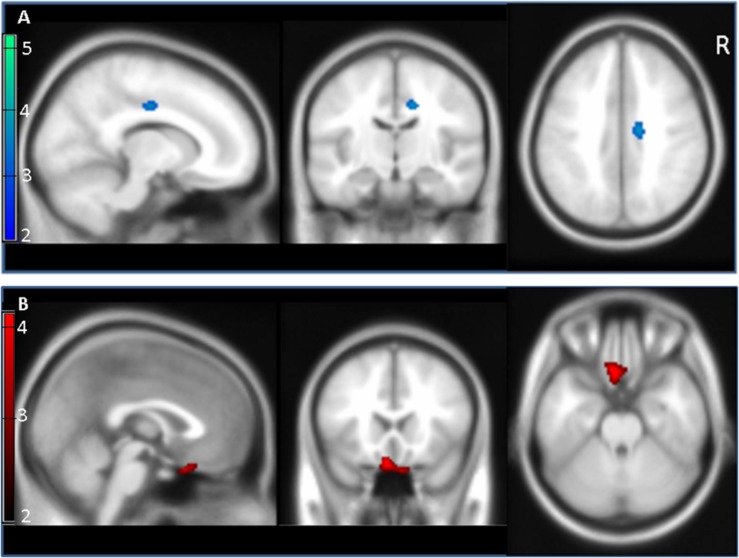
Cerebral opioid receptor binding potential in the entire patient group and after stratification for systemic interleukin-4 (IL-4) expression. **(A)** Patients with fibromyalgia syndrome (FMS) showed reduced opioid receptor binding potential (blue) in mid cingulate cortex (*z* = 3.08) compared to healthy controls (blue area; threshold *p* < 0.005 corresponding to a z-score of 2.58). **(B)** Patients with high IL-4 gene expression showed increased opioid receptor binding potential (red) in fronto-basal cortex (rectal gyrus, *z* = 3.60) compared to FMS patients with low IL-4 expression (red area; *p* < 0.005, *z* = 2.58). Scale bars represent z-scores.

When comparing PET scans of all FMS patients with data of healthy controls (eleven healthy subjects previously scanned using the same acquisition, reconstruction, and quantitation protocols), the opioid binding capacity was lower in patients with FMS in the mid cingulate cortex (MNI coordinates (x,y,z): [14/ – 14/38]; *z* = 3.08, cluster size 66 voxels; threshold: *p* < 0.005; Figure 2A). Using a threshold of *p* < 0.005, we found an increased binding potential in the fronto-basal cortex of FMS patients with higher systemic IL-4 gene expression compared to those with low IL-4 gene expression (rectal gyrus, MNI coordinates (x,y,z): [–6/22/ –24], *z* = 3.60, cluster size 259 voxels; Figure 2B).

## Discussion

We investigated the cerebral opioid receptor availability of FMS patients compared to healthy controls and confirm previous findings showing reduced [18F]FEDPN binding in FMS patients (Harris et al., 2007; Schrepf et al., 2016). Despite the small number of study participants, our data also imply a potential link between IL-4 gene expression and cerebral opioid receptor binding potential.

Opioids exert analgesic effects in only a subgroup of patients with FMS and are not recommended by national and international treatment guidelines as first line medication. A reduction in central opioid receptor binding has been reported in FMS patients particularly in antinociceptive brain regions. Harris et al. investigated 17 patients with FMS compared to 17 healthy controls with PET scans and reported reduced cerebral μ-opioid receptor availability in patients (Harris et al., 2007). The same group later assessed 18 FMS patients and compared pain-evoked functional magnetic resonance imaging data with endogenous μ-opioid receptor binding (Schrepf et al., 2016). The authors observed reduced μ-opioid receptor availability associated with decreased pain-evoked neural activity, suggestive of dysfunction of the endogenous opioid system as one central contributor to FMS related pain. Interestingly, even in our small cohort, we could confirm the finding of reduced cerebral opioid receptor availability in the mid cingulate cortex, and, like in Harris et al. (2007), the findings were lateralized to the right.

Our data on a potential link with systemic IL-4 gene expression levels extend this finding by an interesting neuro-immune connection, which includes a potential role of IL-4 linking the opioid and immune system. IL-4 expression has been investigated in several chronic pain syndromes. In patients with FMS, systemic IL-4 gene and protein expression was reduced compared to controls (Üçeyler et al., 2006; Sturgill et al., 2014). Lower IL-4 levels were found in patients with painful compared to painless polyneuropathies (Üçeyler et al., 2007b) and in complex regional pain syndrome (Üçeyler et al., 2007a). Confirming a role of IL-4 in pain modulation, IL-4 deficient mice are characterized by mechanical hypersensitivity, over-expression of pro-inflammatory cytokines (Üçeyler et al., 2011) and neuronal hyperexcitability (Lemmer et al., 2015). Former (Hao et al., 2006) and recent reports (Eijkelkamp et al., 2016; Oetjen et al., 2017; Bobinski et al., 2018; Nie et al., 2018) strengthen the evidence for a high analgesic potential of IL-4. While systemic pro- and anti-inflammatory cytokine expression does not reflect acute pain intensity, further exploring the link between IL-4 and the opioid system (Kraus et al., 2001) may give important insights into the pathophysiology of chronic pain.

The major limitation of our study is the low number of participants. Two reasons hampered patient recruitment: history or current intake of opioid analgesics, which were exclusion criteria, and the refusal of patients to undergo experimental PET using nuclear tracers. Also, no PET scans were available from the control persons in whom systemic IL-4 levels were investigated, thus [18F]FEDPN binding and its correlation with IL-4 levels could not be assessed in these. Matching of FMS patients and healthy controls was not possible for PET experiments, which is another limitation. We found a cluster in the fronto-basal cortex with a max *z* = 3.6 (259 voxels). Analysis of this cluster showed more voxels in gray matter (101) than in white matter (37). There are two effects which may impact the spatial precision of significant clusters: the limited spatial resolution of the PET images compared to MRI, which is used for overlay to identify the corresponding anatomic structure, and additional smoothing of PET images prior to SPM analysis, which is required to achieve a reasonable statistical power. It is, however, intriguing that differences in the opioid receptor binding potential were found between groups despite this low number of subjects.

## Data Availability Statement

All datasets generated for this study are included in article/Supplementary Material.

## Ethics Statement

The studies involving human participants were reviewed and approved by the Ethics Committees of the Universities of Würzburg and Mainz, Germany. The patients/participants provided their written informed consent to participate in this study.

## Author Contributions

NÜ and CS: study concept and design. NÜ, H-GB, SK, and S-JA: acquisition of data. NÜ, H-GB, MS, FB, and CS: analysis and Interpretation of data.

## Conflict of Interest

NÜ: speaker honoraria and research grants from Biogen, Grifols, Sanofi Genzyme, Shire Takeda. FB: speaker honoraria from Akcea, Pfizer, and Alnylam in the past three years. CS: consulting fees and speaker honoraria from Air Liquide, Akcea, Algiax, Alnylam, CSL Behring, Novartis, Pfizer, Sanofi Genzyme, Takeda, UCB. The remaining authors declare that the research was conducted in the absence of any commercial or financial relationships that could be construed as a potential conflict of interest.

## References

[B1] Baumgartner U. Buchholz H. G. Bellosevich A. Magerl W. Siessmeier T. Rolke R. (2006). High opiate receptor binding potential in the human lateral pain system. *Neuroimage* 30 692–699. 10.1016/j.neuroimage.2005.10.033 16337817

[B2] Bobinski F. Teixeira J. M. Sluka K. A. Santos A. R. S. (2018). Interleukin-4 mediates the analgesia produced by low-intensity exercise in mice with neuropathic pain. *Pain* 159 437–450. 10.1097/j.pain.0000000000001109 29140923 PMC5812806

[B3] Cagnie B. Coppieters I. Denecker S. Six J. Danneels L. Meeus M. (2014). Central sensitization in fibromyalgia? A systematic review on structural and functional brain MRI. *Semin. Arthritis Rheum.* 44 68–75. 10.1016/j.semarthrit.2014.01.001 24508406

[B4] Dehghan M. Schmidt-Wilcke T. Pfleiderer B. Eickhoff S. B. Petzke F. Harris R. E. (2016). Coordinate-based (ALE) meta-analysis of brain activation in patients with fibromyalgia. *Hum. Brain Mapp.* 37 1749–1758. 10.1002/hbm.23132 26864780 PMC6867581

[B5] Eijkelkamp N. Steen-Louws C. Hartgring S. A. Willemen H. L. Prado J. Lafeber F. P. (2016). IL4-10 fusion protein is a novel drug to treat persistent inflammatory pain. *J. Neurosci.* 36 7353–7363. 10.1523/JNEUROSCI.0092-16.2016 27413147 PMC4945660

[B6] Hao S. Mata M. Glorioso J. C. Fink D. J. (2006). HSV-mediated expression of interleukin-4 in dorsal root ganglion neurons reduces neuropathic pain. *Mol. Pain* 2:6. 10.1186/1744-8069-2-6 16503976 PMC1395302

[B7] Harris R. E. Clauw D. J. Scott D. J. McLean S. A. Gracely R. H. Zubieta J. K. (2007). Decreased central mu-opioid receptor availability in fibromyalgia. *J. Neurosci.* 27 10000–10006. 10.1523/JNEUROSCI.2849-07.2007 17855614 PMC6672650

[B8] Harte S. E. Clauw D. J. Hayes J. M. Feldman E. L. St Charles I. C. Watson C. J. (2017). Reduced intraepidermal nerve fiber density after a sustained increase in insular glutamate: a proof-of-concept study examining the pathogenesis of small fiber pathology in fibromyalgia. *Pain Rep.* 2:e590. 10.1097/PR9.0000000000000590 29392206 PMC5741296

[B9] Kraus J. Börner C. Giannini E. Hickfang K. Braun H. Mayer P. (2001). Regulation of mu-opioid receptor gene transcription by interleukin-4 and influence of an allelic variation within a STAT6 transcription factor binding site. *J. Biol. Chem.* 276 43901–43908. 10.1074/jbc.M107543200 11572871

[B10] Lemmer S. Schiesser P. Geis C. Sommer C. Vanegas H. Üçeyler N. (2015). Enhanced spinal neuronal responses as a mechanism for the increased nociceptive sensitivity of interleukin-4 deficient mice. *Exp. Neurol.* 271 198–204. 10.1016/j.expneurol.2015.06.011 26079835

[B11] Nie B. L. Liu C. C. Bai X. H. Chen X. D. Wu S. Y. Zhang S. B. (2018). AKAP150 involved in paclitaxel-induced neuropathic pain via inhibiting CN/NFAT2 pathway and downregulating IL-4. *Brain Behav. Immun.* 68 158–168. 10.1016/j.bbi.2017.10.015 29056557

[B12] Oetjen L. K. Mack M. R. Feng J. Whelan T. M. Niu H. X. Guo C. X. J. (2017). Sensory neurons co-opt classical immune signaling pathways to mediate chronic itch. *Cell* 171:217-228.e13. 10.1016/j.cell.2017.08.006 28890086 PMC5658016

[B13] Schrepf A. Harper D. E. Harte S. E. Wang H. Ichesco E. Hampson J. P. (2016). Endogenous opioidergic dysregulation of pain in fibromyalgia: a PET and fMRI study. *Pain* 157 2217–2225. 10.1097/j.pain.0000000000000633 27420606 PMC5028286

[B14] Sturgill J. Mcgee E. Menzies V. (2014). Unique cytokine signature in the plasma of patients with fibromyalgia. *J. Immun. Res.* 2014:938576. 10.1155/2014/938576 24741634 PMC3987983

[B15] Üçeyler N. Eberle T. Rolke R. Birklein F. Sommer C. (2007a). Differential expression patterns of cytokines in complex regional pain syndrome. *Pain* 132 195–205. 10.1016/j.pain.2007.07.031 17890011

[B16] Üçeyler N. Rogausch J. P. Toyka K. V. Sommer C. (2007b). Differential expression of cytokines in painful and painless neuropathies. *Neurology* 69 42–49. 10.1212/01.wnl.0000265062.92340.a5 17606879

[B17] Üçeyler N. Topuzoglu T. Schiesser P. Hahnenkamp S. Sommer C. (2011). IL-4 deficiency is associated with mechanical hypersensitivity in mice. *PLoS One* 6:e28205. 10.1371/journal.pone.0028205 22164245 PMC3229527

[B18] Üçeyler N. Valenza R. Stock M. Schedel R. Sprotte G. Sommer C. (2006). Reduced levels of antiinflammatory cytokines in patients with chronic widespread pain. *Arthritis Rheum.* 54 2656–2664. 10.1002/art.22026 16871547

[B19] Üçeyler N. Zeller D. Kahn A. K. Kewenig S. Kittel-Schneider S. Schmid A. (2013). Small fibre pathology in patients with fibromyalgia syndrome. *Brain* 136 1857–1867. 10.1093/brain/awt053 23474848

[B20] Üçeyler N. Zeller J. Kewenig S. Kittel-Schneider S. Fallgatter A. J. Sommer C. (2015). Increased cortical activation upon painful stimulation in fibromyalgia syndrome. *BMC Neurol.* 15:210. 10.1186/s12883-015-0472-4 26486985 PMC4618366

[B21] Wolfe F. Smythe H. A. Yunus M. B. Bennett R. M. Bombardier C. Goldenberg D. L. (1990). The American College of rheumatology 1990 criteria for the classification of fibromyalgia. report of the multicenter criteria committee. *Arthritis Rheum* 33 160–172. 2306288 10.1002/art.1780330203

