# Supplementary material for: Cortical Binding Potential of Opioid Receptors in Patients With Fibromyalgia Syndrome and Reduced Systemic Interleukin-4 Levels – A Pilot Study
Source: Front Neurosci. 2020 May 19;14:512. doi: 10.3389/fnins.2020.00512 (PMC7248364; doi:10.3389/fnins.2020.00512)
Supplement: Supplementary file 1 [file Table_1.docx]

**Suppl. Table 1: Study design.**

| **Visits and sites** | **Tasks** | **Time points** |
| --- | --- | --- |
| Visit 1 and 2 (Würzburg) | - Clinical examination - Questionnaires - Blood withdrawal (qRT-PCR) | Baseline + follow up at median of 1.3 years |
| Visit 3 (Mainz) | - PET scan | Following Visit 2 |

**Abbreviations:** PET: positron-emission-tomography; qRT-PCR: quantitative real-time PCR.
