# Supplementary material for: Cortical Binding Potential of Opioid Receptors in Patients With Fibromyalgia Syndrome and Reduced Systemic Interleukin-4 Levels – A Pilot Study
Source: Front Neurosci. 2020 May 19;14:512. doi: 10.3389/fnins.2020.00512 (PMC7248364; doi:10.3389/fnins.2020.00512)
Supplement: Supplementary file 2 [file Table_2.docx]

**Suppl. Table 2: Individual patient data.**

| **ID** | **Age (years)** | **Gender** | **Disease duration (years)** | **NPSI sum score** | **GCPS current pain (NRS)** | **BDI II score** |
| --- | --- | --- | --- | --- | --- | --- |
| PET2 | 51 | M | 20 | 50 | 7 | 30 |
| PET6 | 63 | F | 45 | 15 | 3 | 7 |
| PET9 | 55 | F | 44 | 37 | 4 | 8 |
| PET11 | 58 | F | 28 | 24 | 4 | 17 |
| PET13 | 61 | F | 45 | 20 | 4 | 22 |
| PET17 | 53 | F | 24 | 70 | 7 | 11 |
| PET 28 | 69 | F | 36 | 13 | 6 | 7 |

**Abbreviations:** BDI=Beck Depression Inventory; F=female; FIQ=Fibromyalgia Impact Questionnaire; GCPS=Graded Chronic Pain Scale; M=male; NPSI=Neuropathic Pain Symptom Inventory.
