# Supplementary material for: Cortical Binding Potential of Opioid Receptors in Patients With Fibromyalgia Syndrome and Reduced Systemic Interleukin-4 Levels – A Pilot Study
Source: Front Neurosci. 2020 May 19;14:512. doi: 10.3389/fnins.2020.00512 (PMC7248364; doi:10.3389/fnins.2020.00512)
Supplement: Supplementary file 3 [file Table_3.docx]

**Suppl. Table 3: IL-4 relative gene expression in individual patients.**

| **ID** | **Date V1** | **Date V2** | **Latency V1 to V2 (years)** | **IL-4 V1** | **IL-4 V2** |
| --- | --- | --- | --- | --- | --- |
| PET2 | 28.11.2007 | 20.04.2009 | 1.4 | 1.2 | 1.0 |
| PET6 | 19.12.2007 | 05.06.2009 | 1.5 | 2.2 | 2.0 |
| PET9 | 23.01.2008 | 26.05.2009 | 1.3 | 0.6 | 0.6 |
| PET11 | 08.02.2008 | 10.06.2009 | 1.3 | 0.6 | 0.6 |
| PET13 | 14.03.2008 | 11.05.2009 | 1.2 | 0.9 | 0.9 |
| PET17 | 25.04.2008 | 02.08.2009 | 1.3 | 2.0 | 1.8 |
| PET 28 | none | 31.05.2010 | NA | NA | 0.2 |

**Abbreviations:** NA=not applicable; V=visit.
